# East Meets West: Perspectives on the Development of Chiropractic Education and Profession Under Hong Kong’s “One Country, Two Systems” Policy

**DOI:** 10.7759/cureus.40974

**Published:** 2023-06-26

**Authors:** Andy Fu Chieh Lin, Valerie Chu

**Affiliations:** 1 Chiropractic and Physiotherapy Centre, New York Medical Group (NYMG), Hong Kong, CHN; 2 Research, The Chiropractic Doctors' Association of Hong Kong, Hong Kong, HKG

**Keywords:** chiropractic doctors association of hong kong, hong kong chiropractic college, hong kong chiropractor, hong kong public health, chiropractic

## Abstract

In Hong Kong, chiropractic care developed under the national policy of "one country, two systems," whereby Hong Kong retained its own economic and political systems while remaining part of mainland China. This environment allowed Western education standards and practices to be adopted while integrating local cultural beliefs. In this respect, chiropractic healthcare emerged as an early model of culturally synergistic *East meets West* care. However, despite Hong Kong’s large population and interest in natural health options, the field faces several challenges, including competition with other professions, costs of education, and political uncertainty. Collaborating across professions, demonstrating value through outcomes, and adapting to cultural contexts may facilitate the integration of chiropractic care into Hong Kong’s healthcare system. Moreover, positioning chiropractic care within Hong Kong’s integrative *East meets West *healthcare movement may help sustain the practice regardless of political shifts. Through strategic partnerships and the maintenance of high standards balanced by cultural sensitivity, the chiropractic field in Hong Kong exemplifies the global spread of healthcare professions. Overall, chiropractic care in Hong Kong has had to navigate complex sociocultural and political circumstances and evolve into an integrated approach, reflecting the region’s pluralistic society. The study first discussed the development of the chiropractic profession in Hong Kong under the "one country, two systems" policy. It then examined the opportunities and challenges faced by the profession and concluded by delving into the future prospects of chiropractic in the region.

## Editorial

In Hong Kong, chiropractic care developed under the national policy of "one country, two systems," whereby Hong Kong retained its own economic and political systems while remaining part of mainland China. This environment allowed Western education standards and practices to be adopted while integrating local cultural beliefs. In this respect, chiropractic healthcare emerged as an early model of culturally synergistic *East meets West* care. However, despite Hong Kong’s large population and interest in natural health options, the field faces several challenges, including competition with other professions, costs of education, and political uncertainty. Collaborating across professions, demonstrating value through outcomes, and adapting to cultural contexts may facilitate the integration of chiropractic care into Hong Kong’s healthcare system. Moreover, positioning chiropractic care within Hong Kong’s integrative *East meets West* healthcare movement may help sustain the practice regardless of political shifts. Through strategic partnerships and the maintenance of high standards balanced by cultural sensitivity, the chiropractic field in Hong Kong exemplifies the global spread of healthcare professions. Overall, chiropractic care in Hong Kong has had to navigate complex sociocultural and political circumstances and evolve into an integrated approach, reflecting the region’s pluralistic society. Balancing global consistency with local adaptation, this model can inform the field of chiropractic care across borders and cultures.

Introduction

Following the handover of Hong Kong from the United Kingdom to China in 1997, a national policy of "one country, two systems" was adopted to govern the territory. Under this policy, Hong Kong has been allowed to retain its healthcare systems independent of mainland China. In other words, while Hong Kong is part of China, it retains its own systems-including legislative and judicial systems based on the British model [1]. Consequently, Hong Kong has preserved both capitalist and democratic freedoms while being part of socialist China. Hong Kong’s "one country, two systems" policy provides a unique environment for the development of Western healthcare professions, including chiropractic care [2]. However, despite maintaining Western professional standards, the development of the chiropractic profession in Hong Kong has necessarily involved adapting to local culture and health beliefs. This adaptation has resulted in a chiropractic philosophy unique to Hong Kong, one combining *East and West*. The "one country, two systems" policy created an environment in which such cross-cultural adaptation and integration could occur. Accordingly, this study examines how the "one country, two systems" policy has shaped the development of the chiropractic profession in Hong Kong, including the opportunities and challenges it presents for the profession going forward.

Development of the chiropractic profession in Hong Kong

As a healthcare profession, chiropractic emerged in the late nineteenth century, when it was founded by Daniel David Palmer in the United States [3]. Chiropractic has since evolved into a widely recognized and practiced form of complementary and alternative medicine in the West, one emphasizing the diagnosis, treatment, and prevention of mechanical disorders of the musculoskeletal system, particularly the spine [4]. The chiropractic profession has a long history in Hong Kong, with the practice introduced sometime before the Second World War (1939-1945) [5]. In 1993, the Chiropractors Registration Ordinance was passed to regulate chiropractors and provide title protection, making Hong Kong one of the first countries in Asia to formally recognize chiropractic as a healthcare profession [6]. The Chiropractors Registration Ordinance permitted the adoption of established Western chiropractic standards and Anglocentric education models, without influence from Traditional Chinese Medicine (TCM). As a result, Hong Kong’s chiropractors are recognized by their Western peers. For instance, the Hong Kong Chiropractic College and Education program, founded by Eric Chu in partnership with the McTimoney College of Chiropractic of the United Kingdom in 2023 was approved by Hong Kong Education Bureau [7].

**Figure 1:**
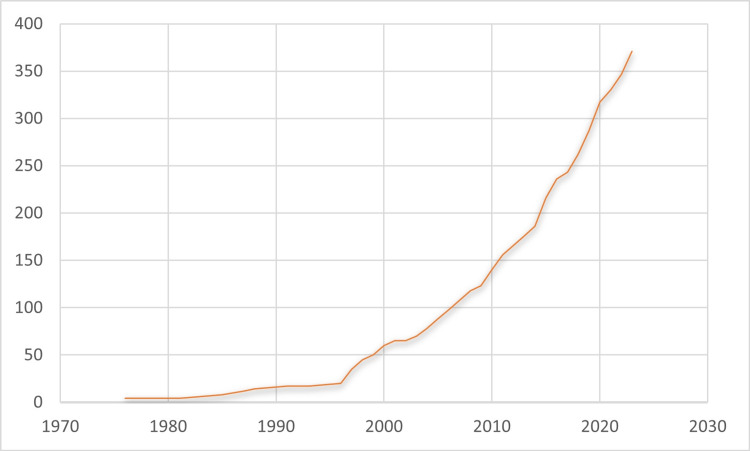
Number of Hong Kong registered chiropractors.

However, despite maintaining Western educational and practice standards, chiropractors in Hong Kong had to adapt their approach to account for local cultural beliefs and health practices. Established by Vincent Chan, Albert Leung, and Alex Shiu in 2000, the Chiropractic Doctors Association of Hong Kong (CDAHK) has sought to promote chiropractic in Hong Kong [8], primarily through public promotion campaigns advancing a model of chiropractic care integrating *East meets West* characteristics [8]. As there was no local education, all CDAHK chiropractors trained in Western educational institutes [9]. As the chairman of the CDAHK (2000-2014), Chan had to familiarize the association with local healthcare concepts and learn how to explain chiropractic in culturally relevant ways to the local population in the beginning. As chiropractors serve local communities in Hong Kong, they have to understand TCM explanations of health and relate these to their knowledge on the relationship between spinal function and well-being. Chu was elected as the chairman (2015-present) and developed the industry with evidence-based, patient-centered, interprofessional, and collaborative approaches [10]. As a result of CDAHK efforts, the number of chiropractors in Hong Kong grew substantially from just four in 1976 to 287 in 2019 [11]. As of April 2023, Hong Kong had a total of 325 registered chiropractors [12] (Figure 1).

To obtain licensure, chiropractors must hold a recognized chiropractic degree and meet the standard of the Chiropractors Council of Hong Kong (CCHK). The scope of practice for chiropractors in Hong Kong generally includes the diagnosis and treatment of chiropractic conditions, ordering and interpreting imaging studies and laboratory tests, and providing nonpharmacological care, such as manual therapies and exercise recommendations [13]. Chiropractic care has gained increasing popularity and recognition in Hong Kong, largely due to its high efficacy in cases where conventional therapies have failed [14] and its low incidence of serious adverse events [15]. Hong Kong’s chiropractic model is focused on ensuring the high professional and education standards seen in Western countries [14] while emphasizing the adaptation of practices to align with the pluralistic Hong Kong healthcare system and address the needs of the local population [2]. This balanced and integrated *East meets West* approach to chiropractic development is a good example of adapting a Western healthcare profession to suit an Asian cultural context. In this regard, the early adoption of legislation and professional standards allowed chiropractic to thrive in Hong Kong, advancing the spread of the profession across Asia [16]. More specifically, by fusing cultural perspectives, Hong Kong's *East meets West* brand of chiropractic provides a model for introducing chiropractic care into other traditional Asian medical systems. Overall, chiropractic in Hong Kong has developed through the adoption of well-established Western structures while accommodating local beliefs. This synthesis highlights both the possibilities and requirements for transplanting a healthcare profession into different contexts across the globe.

Opportunities and challenges under the "one country, two systems" policy

The governing principle of "one country, two systems" presents both significant opportunities and challenges for the development of the chiropractic profession in Hong Kong [2]. In terms of opportunities, Hong Kong’s large population of over 7.4 million people [17] and growing middle-class demographic are increasingly exposed to Western culture, resulting in rising interest in Western and alternative healthcare approaches like chiropractic. Therefore, chiropractors have access to a substantial potential patient base seeking non-pharmaceutical and natural treatment options for the management of various musculoskeletal and neuromuscular conditions, including back pain, neck pain, and headaches [18]. Moreover, the adoption of chiropractic therapy in the sports industries of China and Hong Kong can facilitate participation in international events and attract investment, thereby promoting economic growth and innovation [18]. However, as noted, chiropractic in Hong Kong also faces several challenges. Notably, there is strong competition from established healthcare professions, such as physiotherapy and acupuncture, which have a long history in the region. Although chiropractic care has demonstrated its ability to successfully address complex symptoms while avoiding unwanted treatment side effects [19], chiropractors need to actively set their services apart and convey the advantages of chiropractic care for specific conditions. In the absence of government subsidies, chiropractors also need to cater to local preferences for minimally invasive and natural therapeutic methods.

Hong Kong’s healthcare sector is transforming, with the focus shifting to primary and preventative care and a growing interest in Western and alternative healthcare approaches. Chiropractic has played a significant role in this healthcare transformation by providing patients with alternative approaches to health and wellness care, creating new opportunities in electronic health recordkeeping, and connecting stakeholders within the healthcare system to provide patients with comprehensive care solutions [20]. Patient’s quality of care, treatment outcomes, and experiences can be improved by extending chiropractic care beyond hospital and clinical settings and into the home [21]. While Hong Kong’s status as a global financial center allows for greater investment in research and professional services [22], which will benefit the chiropractic profession, the higher costs of living in the region translate into higher costs for chiropractic education and business operations. As most chiropractors received their educations in Australia (34%), the United States (31%), and Canada (8%) [9], this could limit the growth of the profession by reducing access to chiropractic education and discouraging new chiropractors from practicing. In this respect, partnerships with universities have helped address chiropractic education needs.

There is uncertainty regarding potential changes to Hong Kong’s "one country, two systems" policy in the future, particularly with the policy slated for termination in 2047 [23]. Changes could lead to the separation of Hong Kong’s healthcare system from that of mainland China, a separation maintained by Western professions like chiropractic. To ensure stability, chiropractors must actively coordinate with local governments to produce collaborative research, referrals, education, and policies on chiropractic approaches [24]. In this respect, Dr. Valerie Chu, the chairperson of the HKCC and treasurer of the CDAHK, was appointed to the 2021 Election Committee for the Medical and Health Services Subsector, making her eligible to nominate a candidate for the Legislative Council [24]. As a politically involved chiropractor, Chu has been instrumental in reinforcing the position of chiropractic care in the public health system and enhancing public safety and trust in chiropractic care in the region.

In summary, while the "one country, two systems" policy presents opportunities for chiropractic growth in Hong Kong’s open market, various challenges, including competition, costs, and political uncertainty, also need to be addressed. Given the region’s evolving relationship with China, Hong Kong’s chiropractic profession needs to adopt strategies to advance its position in Hong Kong’s healthcare landscape. Examples of such strategies include increasing public education on chiropractic, integrating chiropractic into the TCM system, appealing to people’s preferences for natural healthcare options, and lobbying for the value of Western healthcare professions.

Prospects of chiropractic in Hong Kong

To meet the complex needs of an aging population, Hong Kong’s healthcare system is shifting toward preventative and primary care [25]. Given its early identification of musculoskeletal issues, reduction of associated risks, and promotion of healthy lifestyles, chiropractic is in an advantageous position to support a healthcare strategy centered on prevention [24]. As such, chiropractic in Hong Kong has a promising future, provided it continues to adapt to local needs and circumstances. Strengthening interprofessional education and partnerships will help chiropractic take advantage of opportunities and overcome challenges in Hong Kong’s complex healthcare environment under the "one country, two systems" policy [2].

Integrating chiropractic into Hong Kong’s larger healthcare system through collaboration and referrals from other professions is essential for its continued success in the region. Indeed, general practitioners (15%) and orthopedic surgeons (14%) frequently refer patients to chiropractors, as do physiotherapists and acupuncturists [9]. Chiropractors also refer their patients to their medical peers, with one survey finding that other chiropractors accounted for 11% of patient referrals [9]. Interprofessional education exposing students in various disciplines to chiropractic will increase professional understanding of chiropractic care and referral potential. Partnerships on research and practice guidelines with organizations such as the CDAHK and Hong Kong Chiropractic College can also aid integration.

Chiropractic in Hong Kong is well positioned for continued growth, providing that it continues to adapt its education and practice to suit local values and the "one country, two systems" policy framework, which maintains the separation of mainland China’s healthcare system and Hong Kong’s pluralistic model [2]. Examples of the successful integration of Western and Eastern medicines in clinical case management in physiotherapy, nursing, and university medical programs point to the potential for interprofessional collaboration with chiropractic in Hong Kong [26-36].

The rise of *East meets West* healthcare and education models that blend Western and Eastern practices, philosophies, and perspectives may provide opportunities for the expansion of chiropractic in Hong Kong. The profession’s status as an *alternative* but evidence-based approach focused on natural, noninvasive care is well-aligned with local preferences and cultural beliefs regarding health. Therefore, positioning chiropractic within the *East meets West* integrative medicine movement may help strengthen its relevance to Hong Kong’s population. However, if substantial changes to Hong Kong’s political system were to lead to its relative separation from mainland China, chiropractic care in the region would face an uncertain future. Therefore, it is important to monitor the region’s "one country, two systems"* policy* and identify any shifts and related impacts. Maintaining high professional standards and continuing to integrate with the local cultural context may provide the best chance of sustaining the chiropractic profession regardless of political circumstances.

Conclusions

Under Hong Kong’s "one country, two systems" policy, chiropractic has navigated a complex sociopolitical environment by adopting Western standards of education and practice while adapting to local cultural beliefs and health practices. Presenting both opportunities and challenges, this fusion of East and West has allowed chiropractic to evolve in Hong Kong. Chiropractic governing bodies and institutions must strengthen the profession by cultivating interprofessional partnerships, producing research demonstrating the value of chiropractic care, and developing culturally synergistic curricula and practice. The profession can also be sustained by closely monitoring any policy changes, ensuring high standards, and adapting to local circumstances. Significantly, the chiropractic profession can secure its future by integrating into Hong Kong’s healthcare system and positioning itself within the *East meets West* movement. Through strategic partnerships and advocacy, chiropractic in Hong Kong can thrive, providing a model of balancing global standards and cultural integration in the dissemination of healthcare professions around the world.
